# Pathology and Therapeutic Significance of Fibroblast Growth Factors

**DOI:** 10.3390/targets3010005

**Published:** 2025-02-02

**Authors:** Oshadi Edirisinghe, Gaëtane Ternier, Thallapuranam Krishnaswamy Suresh Kumar

**Affiliations:** 1 Cell and Molecular Biology Program, University of Arkansas, Fayetteville, AR 72701, USA; 2 Department of Chemistry and Biochemistry, University of Arkansas, Fayetteville, AR 72701, USA

**Keywords:** fibroblast growth factors, FGF, fibroblast growth factor receptors, FGFR, FGF signaling

## Abstract

The fibroblast growth factor (FGF) family includes 22 proteins in humans. Based on their mode of action, there are three families of FGFs: paracrine FGFs (FGF 1–10, 16, 17, 18, 20, and 22), intracrine FGFs (FGF 11–14), and endocrine FGFs (FGF 19, 21, and 23). FGF signaling plays critical roles in embryonic development, tissue repair, regeneration, angiogenesis, and metabolic regulation. They exert their cellular functions by binding, dimerization, and activation of transmembrane FGF receptors (FGFRs). Aberrant FGF signaling is associated with various human diseases. Thus, understanding the unique properties of FGF signaling will help to explore new therapeutic interventions against FGF-mediated pathological conditions. This review will discuss the differential expression and regulation of each FGF under normal human physiological and pathological conditions. Moreover, we will outline current therapeutics and treatment strategies that have been developed against FGF-related pathology.

## 1. Introduction

**Figure 1.**
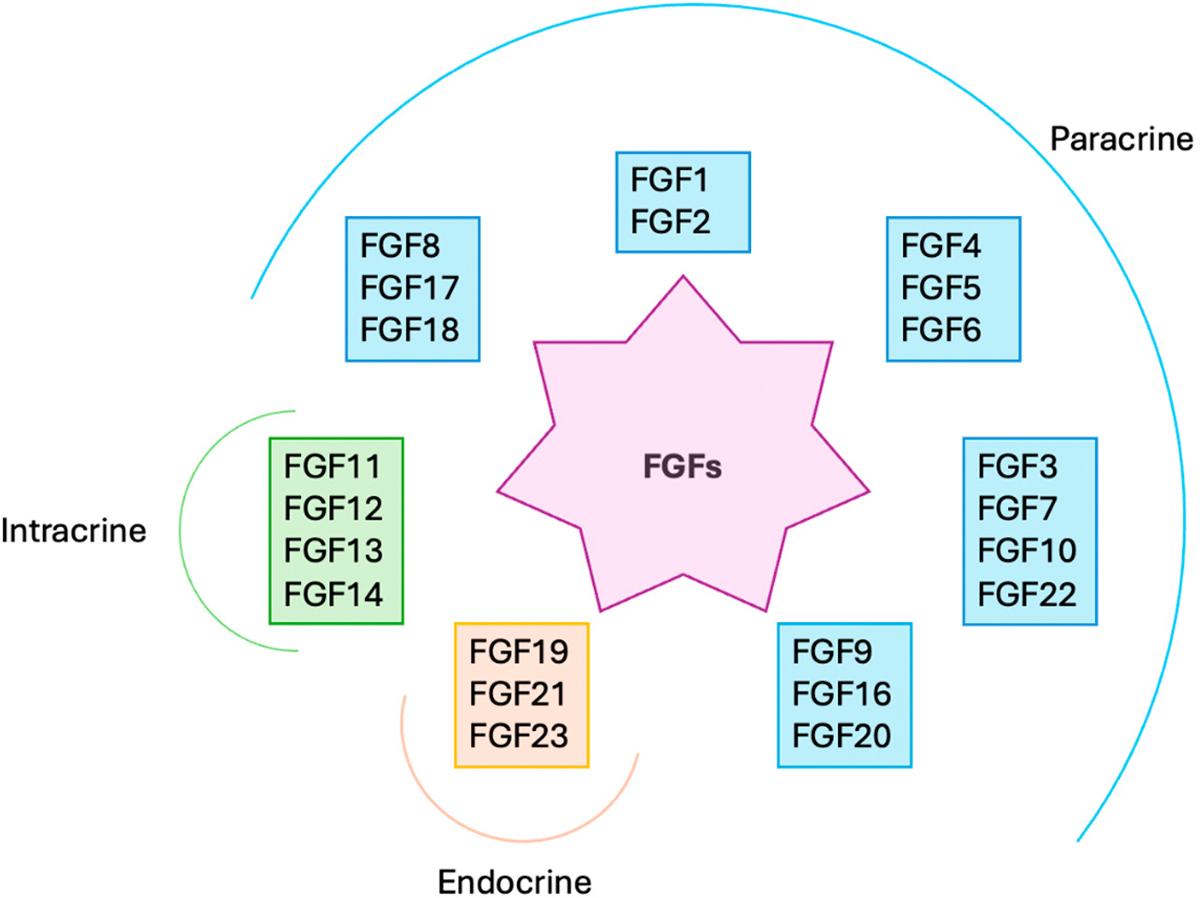
FGF classification based on mode of action. Based on their mode of action, there are three families of FGFs: paracrine/autocrine FGFs (FGF 1–10, 16, 17, 18, 20, and 22), intracrine FGFs (FGF 11–14), and endocrine FGFs (FGF 19, 21, and 23). Based on sequence homology and phylogeny, FGFs can be further divided into subfamilies. There are five paracrine FGF subfamilies, which include the FGF1 subfamily (FGF1 and FGF2), the FGF4 subfamily (FGF4, FGF5, and FGF6), the FGF7 subfamily (FGF3, FGF7, FGF10, and FGF22), the FGF8 subfamily (FGF8, FGF17, and FGF18), and the FGF9 subfamily (FGF9, FGF16, and FGF20). The intracrine subfamily is composed of FGF11, FGF12, FGF13, and FGF14, and the endocrine subfamily corresponds to the FGF19 subfamily (FGF19, FGF21, and FGF23).

The fibroblast growth factor (FGF) family includes 22 proteins in humans. Based on their mode of action, there are three families of FGFs: paracrine/autocrine FGFs (FGF 1–10, 16, 17, 18, 20, and 22), intracrine FGFs (FGF 11–14), and endocrine FGFs (FGF 19, 21, and 23) (Figure 1) [1]. Based on sequence homology and phylogeny, FGFs can be further divided into subfamilies. There are five paracrine FGF subfamilies, which include the FGF1 subfamily (FGF1 and FGF2), the FGF4 subfamily (FGF4, FGF5, and FGF6), the FGF7 subfamily (FGF3, FGF7, FGF10, and FGF22), the FGF8 subfamily (FGF8, FGF17, and FGF18), and the FGF9 subfamily (FGF9, FGF16, and FGF20) [1]. The intracrine subfamily is composed of FGF11, FGF12, FGF13, and FGF14, and the endocrine subfamily corresponds to the FGF19 subfamily (FGF19, FGF21, and FGF23) [2,3]. FGFs regulate many cellular functions, including cell proliferation, differentiation, migration, survival, and metabolism, thereby enabling FGF signaling to play critical roles in embryonic development, tissue repair, regeneration, angiogenesis, and metabolic regulation [1,2]. They exert their cellular functions by binding, dimerization, and activation of transmembrane FGF receptors (FGFRs), which are receptor tyrosine kinases [4,5]. There are four main types of FGFRs: FGFR1–FGFR4. Further, due to alternative splicing, FGFR1–FGFR3 generate multiple isoforms, namely, b (epithelial-specific) and c (mesenchymal-specific) isoforms. Hence, altogether, there are seven major FGFR isoforms (FGFR1b, FGFR1c, FGFR2b, FGFR2c, FGFR3b, FGFR3c, and FGFR4) [1,6]. In paracrine FGFs, FGF–FGFR signaling is mediated by heparan sulfate, which acts as the coreceptor [7]. However, endocrine FGFs use klotho proteins as the coreceptor to exert their biological functions. The α- klotho and β-klotho are the two major types of klotho proteins. Due to their lack of affinity towards heparin, endocrine FGFs join systemic circulation (similar to hormones), unlike paracrine/autocrine FGFs, which exert a localized effect [4]. The FGF–FGFR signaling complex will activate downstream intracellular signaling pathways such as the mitogen-activated protein kinase (MAPK), phosphatidylinositol-3-kinases/serine/threonine kinase (PI3K/AKT), and extracellular signal-regulated kinase 1/2 (ERK1/2) pathways [2,8]. The intracrine FGFs function in an FGFR-independent manner and warrant further research on intracrine FGF-mediated signaling [9]. Aberrant FGF signaling is associated with various human diseases, such as cancer, achondroplasia, FGF-related craniosynostosis, dwarfism syndrome, Alzheimer’s disease, Parkinson’s disease, rickets, chronic kidney disease, diabetes, and obesity [1]. Thus, understanding the unique properties of FGF signaling will aid in the exploration of new therapeutic interventions for different pathologies. This review will discuss the expression and regulation of each FGF under normal human physiological and pathological conditions and the therapeutic applications/strategies based on FGF signaling.

## 2. Paracrine FGFs

### 2.1. FGF1 Subfamily

FGF1 subfamily members are the most studied FGFs in the FGF family. The members include FGF1 and FGF2 (considered as the prototypical ligands of the FGF family). They possess a characteristic β-trefoil core, organized into three copies of a four-stranded β sheet. The amino and carboxy termini extend outward from the core [10]. FGF1 and FGF2 are mitogenic FGFs that mediate cell proliferation, differentiation, and angiogenesis by interacting with different isoforms of FGFRs and heparin sulfate (HS) coreceptors. Both members predominantly exert their biological functions in a paracrine fashion; however, recent studies have reported an autocrine mode of action as well. They exert a plethora of biological functions during embryogenesis and adulthood, which will be extensively discussed in the following section [7,11–13].

#### 2.1.1. FGF1

The *Fgf1* gene is located on chromosome 5q31.3 [14]. The FGF1 mature peptide is made of 155 amino acids. Due to its low isoelectric point, FGF1 is also known as the acidic fibroblast growth factor. Moreover, due to its ability to bind to all FGFR isoforms, FGF1 is considered the universal ligand of the FGF family [10]. It is widely expressed and plays a significant role during embryogenesis, coordination of neural tube development, organogenesis (e.g., heart, lungs, kidneys, skeletal derivatives, and so forth), and limb regeneration [7]. Due to its inherent ability to stimulate cell proliferation, angiogenesis, and differentiation, FGF1 is associated with wound healing [15,16]. Further, the anti-apoptotic activity of FGF1, which promotes cell survival, is proven by many studies [17,18]. Contradicting the long-held notion of FGF1 as a paracrine mediator, novel studies report that FGF1 can also act in an autocrine manner to deliver metabolic effects. FGF1 derived from adipose tissues is reported to be associated with insulin sensitization, glucose uptake, and adipose remodeling [19]. Huang et al. (2017) reported that the FGF1 mutants with reduced HS binding had reduced mitogenicity while inducing metabolic effects in vivo. They further postulated that the stability of the FGF1–FGFR complex will be the key determinant that decides the mode of action of FGF1 (i.e., mitogenic vs metabolic) [20]. These findings can be explored further to develop therapeutics against type 2 diabetes and obesity.

Tejedor et al. (2024) reported that topically administered FGF1 and vascular endothelial growth factor A (VEGF-A) mRNA through lipid nanoparticles promoted microvascular sprouting in mice, leading to faster wound closure in diabetic mice, suggesting a potential therapy to treat diabetic foot ulcers [21]. Forouzanfar et al. (2018) showed that the FGF1 gene transfected into mesenchymal stem cells derived from adipose tissues improved neuropathic pain due to chronic constriction injury by reducing inflammation [22]. Further, adeno-associated virus-mediated delivery of FGF1 gene transfer enhanced endothelial progenitor cell proliferation and survival, resulting in neovascularization in a pig model for chronic myocardial ischemia and suggesting a potential therapeutic approach that can be further developed in humans [23]. He et al. (2019) reported that the fabrication of heparin-immobilized silk fibroin hydrogels loaded with FGF1 on wounds led to an acceleration of the wound-healing process in rats [24]. A similar delivery method was adopted by Guan et al. (2022) with FGF1-loaded sericin hydrogels to treat endometrial injury and fibrosis. The hydrogel treated with FGF1 enhanced the migration and infiltration of endometrial stromal cells, restored the thickness of the uterine wall, and increased the abundance of uterine glands after endometrial injury. Sustained treatment with FGF1 restored the fertility of rats [25]. In a different context, FGF1 exerted protection during acute hepatotoxicity due to acetaminophen overdose by suppressing apoptosis of hepatocytes, reducing inflammation and oxidative and endoplasmic reticulum stress [26]. Huang et al. (2017) reported that an FGF1 mutant (K127D/K128E/K133V) with reduced heparin binding ability (FGF1^ΔHBS^) could be applicable to treat cardiac ischemia-reperfusion injury and improve myocardial infarction in rats by reducing the size of the infarction, restoring the contractile and relaxation rhythms and post-ischemic recovery [27]. The same triple mutant of FGF1 with reduced heparin binding (FGF1^ΔHBS^) conferred protection against cardiotoxicity induced by Adriamycin during chemotherapy via decreasing apoptosis and oxidative stress in primary cardiomyocytes [28]. Further, FGF1^ΔHBS^ inhibited oxidative stress and inflammation in podocytes of diabetic mice. This suggests that FGF1^ΔHBS^ could be a potential treatment option for diabetic nephropathy and chronic kidney disease [29]. Moreover, FGF1 showed a protective role during acute lung injury by inhibiting cell apoptosis, inflammation, and oxidative stress [30]. Further, the design of chimeric proteins with FGF1 has been reported in the literature. FGF1/CPP-C chimeric protein was reported to protect the intestine during carbon ion radiotherapy in pancreatic carcinoma [31]. The engineering of paracrine–endocrine FGF chimeras has also been reported. Zhao et al. (2019) published that the FGF1^ΔHBS^–FGF21^C-tail^ chimera (composed of an FGF1 mutant with reduced heparin binding ability attached to the C-terminus of FGF21) showed enhanced thermal stability and in vivo half-life with elevated glycemic control and improved insulin resistance [32].

**Figure 2.**
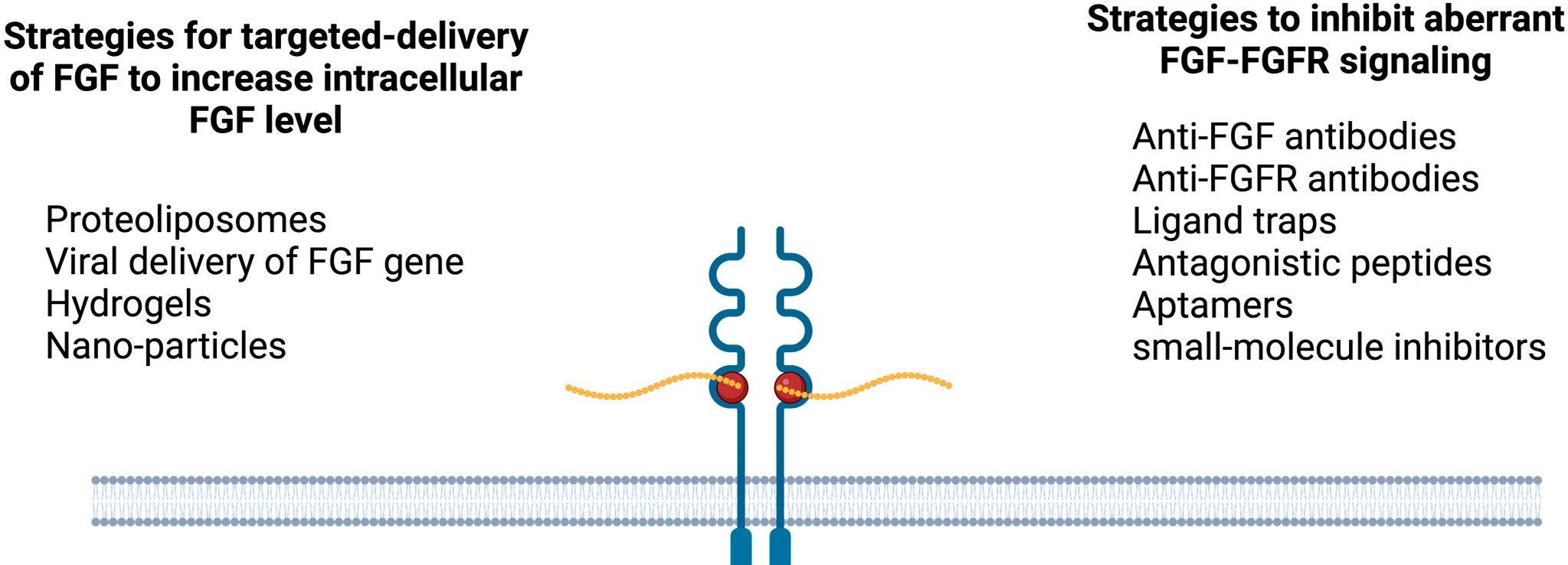
Strategies to optimize FGF–FGFR signaling in therapeutic applications. Dysregulation of FGF levels (i.e., overexpression or under-expression) is reported to be associated with many disorders. Hence, strategies need to be developed to inhibit/counteract FGF overexpression, or if the FGF level is not sufficient, targeted delivery methods need to be optimized. Figure 2 was generated using Biorender.com (https://biorender.com/ (accessed on 20 October 2024)).

**Figure 3.**
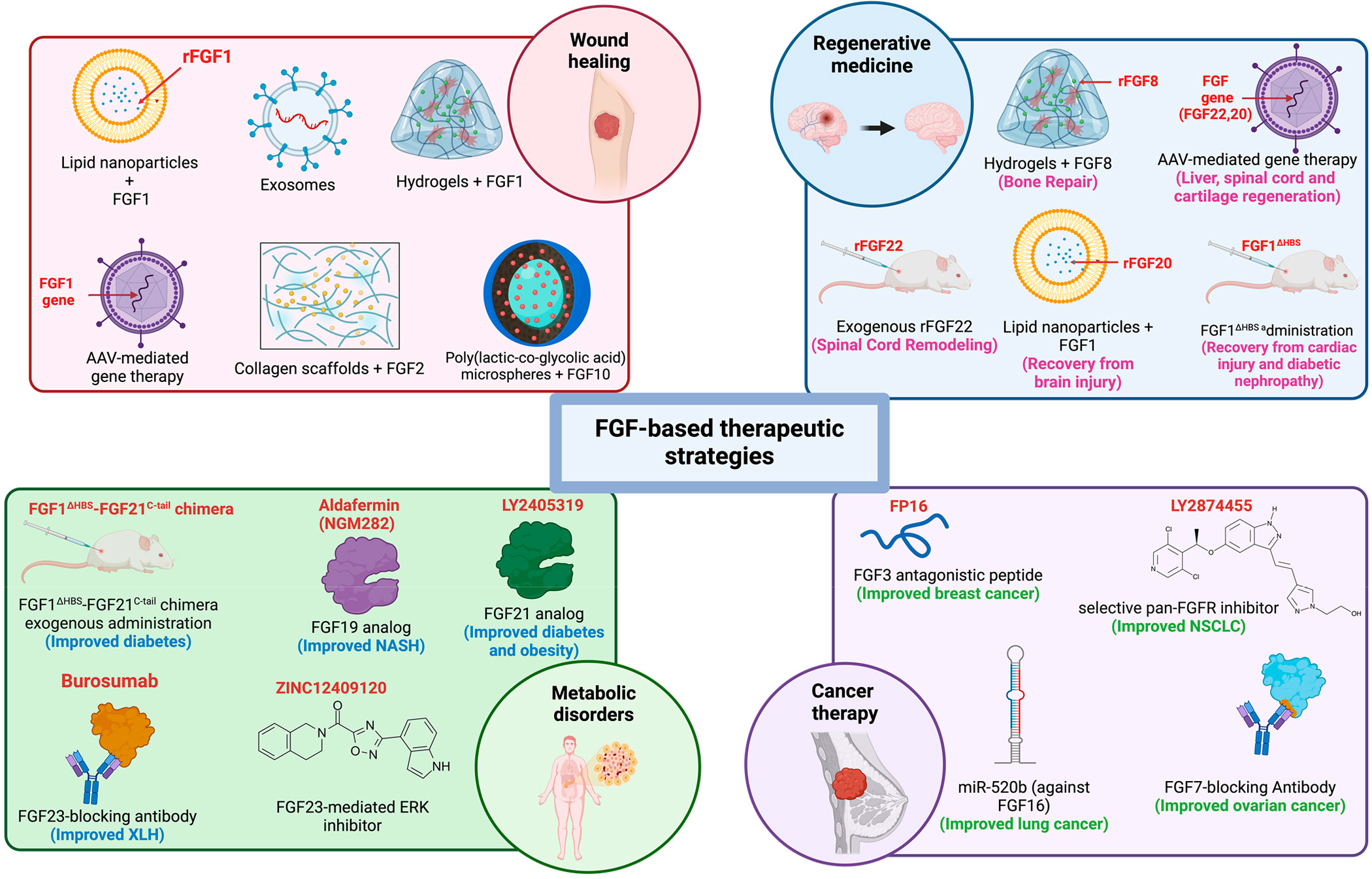
FGF-based therapeutic strategies against wound healing, tissue injury, metabolic disorders, and cancer. Alterations of FGF levels can cause many disorders, including (but not limited to) wound healing, tissue injury, metabolic disorders, and cancer. Lipid nanoparticles, gene therapy, hydrogels, exosomes, and nanoparticles charged with FGFs can be effective for targeted drug delivery. On the other hand, exogenous administration of FGFs will help tissue repair and regeneration during injury. FGF-based chimeric proteins, anti-FGF antibodies, FGFR-inhibitory antibodies, small molecule inhibitors, antagonistic peptides, and microRNA-mediated FGF-gene silencing are well reported in the literature to alleviate disorders/conditions caused by FGF overexpression. Moreover, FGF analogs have been developed to mitigate disorders or complications due to FGF deficiencies. AAV: adeno-associated virus; rFGF: recombinant FGF; FGF1^ΔHBS^: FGF1 mutant with reduced heparin affinity; NASH: non-alcoholic steatohepatitis; XLH: X-linked hypophosphatemia; ERK: extracellular signal-regulated kinase. Figure 3 was generated using Biorender.com (https://biorender.com/ (accessed on 28 October 2024)).

Overexpression of FGF1 is associated with non-small-cell lung cancer [33], colorectal cancer [34], gastric adenocarcinoma [35], thyroid carcinoma [36], breast cancer [37], ovarian cancer [13], pancreatic cancer [31], and many other types of cancer. Small molecule inhibitors against FGFRs, antibody or antagonistic peptide-mediated inhibitors, and FGF ligand traps have been developed to alleviate the detrimental effects of FGF1 overexpression that can lead to FGFR overstimulation [38]. Further, several microRNAs that can exert RNA interference effect during FGF1 transcription have been studied (e.g., miR-143–3p can improve hepatocellular carcinoma by targeting the FGF1 gene) (Figures 2 and 3) [39].

#### 2.1.2. FGF2

FGF2, encoded on chromosome 4 (4q27) [40], is also known as basic FGF and considered the prototypical ligand in entire FGF family members. Heparin proteoglycans act as a coreceptor for FGF2 [41]. FGF2-mediated biological functions are tissue-specific and are mediated via a paracrine (occasionally autocrine) manner [42]. FGF2 is a potential therapeutic option for wound healing, cardiovascular diseases, and nervous system disorders [43]. It has a direct involvement with tumor growth since it can promote cell proliferation, angiogenesis, and metastasis. FGF2 has recently been recognized as a critical regulator in the auditory development [44]. Upregulation of FGF2 is associated with squamous lung cancer [45], non-small-cell lung cancer [46], hepatocellular carcinoma [47], asthma, and chronic obstructive pulmonary disease [42]. Upon ligament and tendon injuries, FGF2 expression is significantly elevated, conferring a protective effect against cartilage repair and healing tendon/ligament injuries [48]. Conversely, downregulation of FGF2 expression is reported to be associated with pathological conditions such as depression and osteoarthritis [49].

Owing to its significant role in neovascularization that promotes tumorigenesis, Fan et al. (2014) developed an antagonistic peptide for FGF2 (named P8), which successfully reduced FGF2-mediated tumor cell proliferation without cytotoxic consequences [50]. The anti-viral drug cidofovir attenuated tumor growth by enhancing apoptosis while inhibiting FGF2-mediated cell proliferation in vascular tumors [51]. Moreover, targeted inhibition of FGF2 by RBM-007 aptamer reduced dysregulated neovascularization in blinding diseases such as age-related macular degeneration, providing promising results [52]. On the other hand, to induce the angiogenic potential of FGF2 in ischemic heart disease and diabetic foot ulcers, collagen-heparin scaffolds with FGF2 (and VEGF) were developed [53]. Boye et al. (2022) reported that FGF2 gene electrotransfer using biocompatible collagen scaffolds to skin improved tendon injuries by stimulating angiogenesis [54].

### 2.2. FGF4 Subfamily

The FGF4 subfamily is composed of FGF4, FGF5, and FGF6. All members act in a paracrine manner by interacting with FGFRs. They are key modulators in cell proliferation and differentiation and play pivotal roles during embryogenesis [55–57].

#### 2.2.1. FGF4

FGF4 is highly expressed in the embryo, which ensures proper limb development, cell proliferation, and pluripotency [55,56,58,59]. Knockout of the *fgf4* gene results in embryonic death [55]. FGF4 was thought to be responsible for maintaining sonic hedgehog (SHH) expression in the zone of polarizing activity ( ZPA), forming a feedback loop where SHH and FGF4 mutually reinforce each other to ensure proper limb bud growth and patterning [55,56]. More recent studies challenge this model by demonstrating that in mouse limbs, even when FGF4 is inactivated, SHH expression continues, and limb development progresses normally, which suggests that other FGFs, FGF8 in particular, would be able to compensate for the loss of FGF4 [55,58]. Furthermore, the importance of FGF4 in liver diseases such as autoimmune hepatitis (AIH) and non-alcoholic fatty liver disease (NAFLD) has recently grown in interest [60–62]. Lin et al. (2024) demonstrated that FGF4 promotes a shift from pro-inflammatory M1 to anti-inflammatory M2 macrophages, resulting in decreased liver inflammation and necrosis [61]. FGF4 would also inhibit ferroptosis, a form of cell death, protecting liver cells from oxidative damage and cell death [60].

A study by Song et al. showed that not only does FGF4 increase lipid oxidation and glucose tolerance in the liver, but it also reduces inflammatory damage [62]. Loss of FGF4 in the liver worsens hepatic steatosis and liver damage in mice fed a high-fat diet, while recombinant FGF4 administration significantly reduces liver fat accumulation, inflammation, and fibrosis. The protective effects of FGF4 in diet-induced non-alcoholic fatty liver disease (NAFLD) are dependent on FGFR4 activation in the liver, which triggers a Ca2+/calmodulin-dependent protein kinase kinase beta (CaMKKβ) and AMP-activated protein kinase (AMPK) signaling cascade, thus enhancing fatty acid oxidation and reducing hepatocellular apoptosis and liver damage [62].

Similarly to several other members of the FGF family, FGF4 would also have wound-healing properties, as demonstrated by Sun et al. in a recent study [63]. The protein promotes re-epithelialization through the activation of the p38 MAPK–GSK3β pathway, stabilizing the slug protein and triggering epithelial–mesenchymal transition (EMT) in keratinocytes [63]. Despite the aforementioned beneficial effects, overexpression of FGF4 is associated with hepatocellular carcinoma [64], urinary bladder cancer [65], ovarian cancer [66], lung adenocarcinoma [67], and breast cancer [68].

Considering the beneficial effects of FGF4, several therapeutics have been developed. Flynn et al. (2008) reported a phase 3 clinical trial testing the efficacy of Alferminogene tadenovec, a potential drug that delivers FGF4 via gene therapy to increase angiogenesis to alleviate myocardial ischemia in women [69]. Further, intravenous administration of phagocytes transfected with FGF4 gene with biodegradable gelatin complex improved angiogenesis in rats with myocardial ischemia [70]. More recently, Liang et al. (2022) reported that exosomes derived from mmu_circ_0001052-adipose-derived stem cells increased angiogenesis by stimulating FGF4–p38/MAPK signaling expression and improved wound healing in diabetic foot ulcers in mice [71].

#### 2.2.2. FGF5

FGF5, whose gene is located on chromosome 4 (4q21.21) [72], is expressed in the root sheath, where it regulates hair growth by intervening at the late stage of the anagen phase [73]. The role of FGF5 and its mutations in hair growth has been studied in several species, such as rabbits, dogs, mice, and sheep [73–75]. Alterations of the *FGF5* gene lead to the angora phenotype in mice and trichomegaly in humans [76]. The angora phenotype would be caused by a deletion principally on exon 1 of *Fgf5* [73,74]. Trichomegaly is attributed to a Y174H missense mutation resulting in a weaker and unstable affinity with FGFR1, negatively altering the FGF5 regulation of hair growth [73,76]. FGF5 is constituted of 268 amino acids but can be subject to alternative splicing where exon 2 is skipped and produces a 123 amino acids protein, FGF5S. FGF5S also acts as an FGF5 antagonist, as it can bind to FGFR1 and inhibit the binding of FGF5 to its receptor, thus preventing hair growth regulation [73]. FGF5 does not only affect hair length but also density and thickness, based on studies performed on sheep. A recent study by Xu et al. underlines the possible effect of gene-edited FGF5 in increasing secondary hair follicle density (SHF) through Wnt/β-catenin signaling, as well as a decrease in cortisol levels, thus alleviating stress [75]. The role of FGF5 in hair growth regulation makes the protein a promising candidate for treating hair loss. In addition to its involvement in hair growth, muscle development, and lung cancers [77,78], an increase in muscle mass was observed in a myostatin (MSTN) FGF5 double knockout sheep model, which may be of great interest to the meat industry [77].

In the literature, monoterpenoid family members (botanically derived compounds) [79] and intradermal injection of siRNA targeted for FGF5 modified by cholesterol [80] are reported to be effective in managing hair loss due to their ability to induce hair growth.

#### 2.2.3. FGF6

FGF6, also named HST2, is expressed in skeletal muscles, where it promotes myogenesis and angiogenesis [81]. Studies on mice models have suggested that FGF6 is necessary for proper healing and muscle regeneration [81–84]. Following an injury, FGF6 promotes muscle regeneration by activating the ERK1/2 pathway through binding with FGFR1 and FGFR4 to increase the expression of cyclin D1, myogenin, and MyoD [83,84]. FGF6 appears to indirectly upregulate the expression of MyoD by activating muscle stem cells, which will enter the cell cycle and subsequently express MyoD [84]. The protein may also alleviate muscle atrophy following nerve injury by increasing the formation of fast-twitch muscle fibers (MyHC-IIb) [82,83].

FGF6 also plays a protective role in myocardial infarction (MI) by inhibiting the Hippo pathway, thus promoting the nuclear accumulation of yes-associated protein (YAP), which in turn [84] facilitates cardiomyocyte (CM) re-entry into the cell cycle, leading to enhanced cardiac repair after MI [85]. By stimulating the expression of cyclin D1 and cyclin E1, FGF6 promotes the progress of CM from the G1 to the S phase of the cell cycle [85]. Lastly, it is important to note that overexpression of FGF6 may lead to prostatic and bladder cancers, while mutation of the gene may disrupt iron metabolism [81]. Armand et al. (2003) reported that injecting human recombinant FGF6 promoted soleus regeneration during myogenesis in mice (*Fgf6*^−*/*−^) by stimulating both proliferation and differentiation [86].

### 2.3. FGF7 Subfamily

The FGF7 subfamily consists of four paracrine FGFs, namely, FGF3, FGF7, FGF10, and FGF22. These members are involved in embryogenesis, organogenesis, wound healing, tissue regeneration, and cell signaling pathways, displaying a plethora of functional diversity [87]. FGF7 subfamily members are exclusively expressed in the mesenchyme and exert their paracrine actions by interacting with epithelial-specific “b” isoforms of FGFRs, mainly via FGFR2b and, to a lesser extent, through FGFR1b. FGF–FGFR signaling requires HS coreceptor interactions [87].

#### 2.3.1. FGF3

FGF3 is encoded on chromosome 11q13.3 [88]. The secreted form of FGF3 contains 239 amino acids, whereas a 273 amino acid residue-long form can be found in both the nucleus and cytoplasm [89]. The association of FGF3 in ear development has been extensively studied. More specifically, FGF3 is involved in inner ear development in many vertebrate species, including chicken [90], mouse, *Xenopus*, and zebrafish [91]. On the same note, the involvement of FGF3 in the optimal coordination of brain development [92], cardiovascular development [93], neural development [94], jaw [95] and ceratobranchial cartilage development [96], and ventral head skeleton development during embryogenesis [97] is reported in the literature.

Contrary to the beneficial effects of FGF3, it is also associated with diseases. Notably, ear malformations and auditory impairments are associated with FGF3 mutation. In a genomic study conducted in 10 unrelated families, FGF3 Lue6Pro (17T>C) and Ile85Met (254delT) homozygous mutations were associated with deafness due to inner ear malformations. Further, a study based on a Somali family (*n* = 9) identified another FGF3 homozygous mutation, R95W, that resulted in inner ear malformation [98]. Overexpression of FGF3 is reported to be associated with cancer. Li et al. (1998) found that FGF3 is associated with angiogenesis and human Kaposi’s sarcoma [99], hepatocellular carcinoma (HCC) metastasis and recurrence of cancer [100], spontaneous breast cancer [101], non-small-cell lung carcinoma (NSCLC) [102], and Laryngeal squamous cell carcinoma [103].

Since FGF3 has been reported to be overexpressed in many cancers, pharmacological inhibition of FGF3 will be beneficial. Wang et al. (2015) developed an FGF3 antagonist peptide, FP16 (VLWLKNR). This peptide was successful at abrogating FGF3–FGFR2 interactions and subsequently inhibiting FGF3-mediated cell proliferation [104]. Hence, FP16 would be a potential treatment option to treat cancer. In NSCLC, a highly selective pan-FGFR inhibitor, LY2874455, combined with abemaciclib (CCND1/CDK4 inhibitor) and gefitinib (tyrosine kinase and epidermal growth factor inhibitor) showed significant improvement in patients with FGF3/FGF4/FGF19/CCND1 amplification on chromosome 11q13 [102]. Retinoic acid [105–107] and follistatin [108] are reported in the literature as mediators that can downregulate FGF3 expression.

#### 2.3.2. FGF7

FGF7 is encoded on chromosome 15q15.21 [109], and its biological activities are solely mediated by FGFR2b–HS interaction in a paracrine fashion. It is also known as keratinocyte growth factor or KGF [110]. FGF7 is involved in keratinocyte proliferation, angiogenesis, and maintenance of inflammatory response during the cutaneous wound healing/repair process [111,112]. Richardson et al. (2009) showed that murine FGF7 and epidermal growth factor (EGF) work in unison during hair follicle morphogenesis by stimulating the development of interfollicular epidermis [113].

On a positive note, the overexpression of FGF7 improved myocardial infarction by reducing mitochondrial oxidative stress via regulating nuclear factor erythroid 2-related factor 2 (Nrf2) and hexokinase 2 levels [114]. Further, Liu et al. (2024) reported a similar redox stress alleviation mediated by FGF7 in osteoblasts, enhancing viability and osteogenic differentiation in vitro [115]. Moreover, Poudel et al. (2017) reported that local delivery of FGF7 has a beneficial effect on bone formation and alleviating mandible defects in rats [116]. FGF7 is reported to be effective in liver regeneration by inducing the proliferation of adult liver progenitor cells during liver injury in mice [117]. Moreover, FGF7 was able to enhance hepatic stellate cells during liver regeneration [118]. The protective role of FGF7 in hepatocytes is further supported by FGF7-mediated downregulation of cholesterol 7α-hydroxylase (CYP7A1) gene expression in hepatocytes, leading to the inhibition of toxic bile acid accumulation in the liver [119].

Owing to the beneficial role of FGF7 during liver regeneration, FGF7-releasing galactosylated poly(DL-lactide-co-glycolic acid) particles targeted to the liver improved liver cell proliferation and hepatic metabolism in diabetic mice [120]. Importantly, human recombinant FGF7, Palifermin (Kepivance^™^), was approved by the FDA to treat severe oral mucositis due to autologous hematopoietic cell transplantation [121] and gastrointestinal mucositis during chemotherapy [122–126].

On the other hand, Jafari et al. (2018) identified novel single-domain antibodies against FGF7 by adopting phage display technology, which could be vastly beneficial in clinical practice for treating diseases arising from FGF7 overexpression [127]. The inhibition of FGF7 overexpression by abolishing its gene expression is widely cited in the literature. Zhang et al. (2019) reported that the development of siRNA that targeted FGF7 was successful in rats, treating choroidal neovascularization in macular degeneration in older people [128]. Similarly, the use of miR381–3p was proven to be effective in cervical cancer [129], miR-455–3p-1 was shown to be effective in pulmonary arterial hypertension through FGF7–FGFR2b–RAS–ERK signaling inhibition [130], and miR-199a-3p was effective against diabetic retinopathy in rats by inhibiting FGF7–FGFR2b–epidermal growth factor–PI3K–AKT signaling [131]. Lastly, Feng et al. (2024) showed that pharmacological inhibition of cancer-associated fibroblast-derived FGF7 through neutralizing antibodies reduced the detrimental effects mediated by FGF7 during ovarian cancer [132].

#### 2.3.3. FGF10

FGF10 is encoded on chromosome 5p12–p13 [133] and interacts with FGFR2b and heparin to exert its biological functions. The mature peptide is composed of 208 amino acid residues. It plays a critical role in glandular morphogenesis, development, patterning, and correct positioning [134–136]. Further, FGF10 involvement in lung development is well-established in the literature. Volckaert et al. (2013) reported that mesenchyme-derived FGF10 is required for the formation of lung buds and the mediation of proximal–distal differentiation [137]. FGF10–FGFR2b signaling is essential for alveolar development and branching of epithelial tubules [138]. Moreover, FGF10 is associated with thalamocortical axon navigation in chicks [94], tooth development in mice [139], face formation during embryogenesis [138], and prostatic growth in rats [140]. These findings underscore the importance of FGF10 in organ development.

The association of FGF10 with diseases is well reported in the literature; most diseases reported are glandular defects, lung disorders, facial defects, and cancer. Lacrimo-auriculo-dento-digital syndrome (LADD) and aplasia of the lacrimal and salivary glands (ALSG) are rare genetic disorders that arise due to FGF10 gene defects [138], exhibiting eye infections, dental caries, atresia, or hypoplasia of lacrimal and salivary glands [141–143]. The 577C>T mutation in *Fgf10* that leads to premature stop codon integration (Arg193stop), the 467T>G mutation that leads to Ile156Arg, and the 409A>T mutation that leads to Lys137stop are significantly associated with LADD and ALSG [141]. Further, FGF10 genetic alterations were associated with lethal lung developmental disorders [144,145], chronic obstructive pulmonary disease [146], bronchopulmonary dysplasia [138], and cleft lip and palate syndrome [138]. Owing to its critical role during embryogenesis and development, certain types of cancer are associated with FGF10, primarily prostate cancer, breast cancer, cholangiocarcinoma [147,148], and gastric cancer [138,149].

In cholangiocarcinoma, FGF10–FGFR2b signaling facilitates tumor cell migration. Oeurn et al. (2023) highlighted that treatment of KKU-M213A cells with an FGFR inhibitor, infigratinib, suppressed FGF10–FGFR2b mediated tumor metastasis by downregulating FGF10–FGFR2b–Akt–mTOR and FGF10–FGFR2b–VEGF–slug pathways [148]. FGF10 can stimulate epithelial cell proliferation, migration, and differentiation and, hence, could provide therapeutic benefits in wound healing. Xu et al. (2020) developed FGF10-loaded poly (lactic-co-glycolic acid) microsphere particles to promote sustained secretion of FGF10 at the wound site. This was proven to be effective since these FGF10-loaded microspheres promoted wound repair by attenuating ER stress in mice [150].

#### 2.3.4. FGF22

FGF22 is encoded on chromosome 19p13 [151]. The structure of FGF22 has not been published thus far. In the published literature, FGF22 is reported as a mitogen that works predominantly in a paracrine manner (autocrine mode of action has also been reported) and as a key player in the development of the embryonic nervous system and maintenance of post-natal and even adult-stage homeostasis. Terauchi et al. (2010) reported that FGF22 is involved in glutamate-mediated excitatory presynaptic ends organization of CA3 pyramidal neurons in the hippocampus [152]. Further, Umemori and Sane (2008) reported that FGF22 stimulated the branching of neurites during presynaptic organization [153]. Moreover, FGF22 is associated with the organization of retinogeniculate synapses [154] and inner hair cell ribbon synapses in the cochlea [155]. Pasaoglu and Schikorsk (2016) showed that FGF22 is involved in the long-term regulation of presynaptic structure. FGF22 plays a critical role during brain development. Presynaptic organization and differentiation, aggregation of synaptic vesicles, and their branching mediated by FGF22 are critical for brain development [156]. Mammalian CA3 pyramidal neuron-derived FGF22 can induce insulin-like growth factor 2 for the stabilization and organization of presynapses in mouse hippocampus [157]. Further involvement of FGF22 in growth plate development of the proximal tibia (postnatal) in rats has been reported [158].

FGF22 expression alterations are affiliated with different types of cancer, such as ovarian cancer [159], lung adenocarcinoma [160], and pancreatic cancer [161]. Due to its predominant association with neural development, FGF22 is involved in auditory defects such as deafness and hearing impairments. A knockout mouse model with FGF22 deletion displayed defects in ribbon synapses of inner hair cells, leading to hearing loss [162]. Further, the deletion of FGF22 led to a reduction in excitatory synapses in the adult mouse hippocampus, which resulted in alterations in affective and cognitive behavior, anxiety levels, and depression-like phenotype [163], suggesting a protective role against psychiatric disorders. Moreover, Xu et al. (2017) published that FGF22 conferred protection against depression by downregulating IL-1β levels in patients with depression [164].

In light of potential clinical applications of FGF22, Jacobi et al. (2015) reported that FGF22 can promote spinal cord remodeling upon injury through synapse formation through FGF22–FGFR1b and FGF10–FGFR2b interactions [165]. Further, mice treated with FGF22 showed less ER stress, lower apoptosis of neuronal cells, enhanced neuron abundance, and axon regeneration during recovery from spinal cord injury [165,166]. Targeted delivery of FGF22 via adeno-associated virus-mediated gene therapy enhanced rewiring and plasticity of motor circuits during spinal cord remodeling after injury. However, the beneficial effects of the intervention are highly dependent on a narrow therapeutic window. Studies need to be optimized to extend the therapeutic window and widen the efficacy and success of the method [167]. Furthermore, FGF22 signaling via FGFR1b and FGFR2b (together with FGF7 and 10) stimulated the proliferation and survival of A6-expressing hepatocytes via the AKT–β-catenin pathway. This pathway was effective against liver injury and promoted liver regeneration in mice [168]. On the other hand, pharmacological inhibition of myocyte enhancer factor 2D via gene therapy (AAV-shMEF2D) led to increased FGF22 expression in a murine ototoxic model. The increased levels of FGF22 facilitated calcium influx into the inner hair cells and promoted ribbon synapse formation [169]. Alternatively, treating mice cochlea with recombinant FGF22 alleviated hearing loss impaired by gentamycin through the maintenance of ribbon synapse abundance [155].

### 2.4. FGF8 Subfamily

The FGF8 subfamily consists of FGF8, FGF17, and FGF18. It plays a crucial role during brain and neuron development, tissue repair, and cell differentiation, predominantly acting in a paracrine manner with interacting HS [170].

#### 2.4.1. FGF8

FGF8 is expressed in several tissues, where it ensures embryogenesis and morphogenesis, especially in body axis elongation, limb development, and midbrain and hindbrain formation [171–173]. FGF8 possesses multiple isoforms, of which FGF8a and FGF8b may be the most active, especially in brain development [171,172]. FGF8 negatively affects bones by binding to FGFRL1, FGFR2IIIc, FGFR3IIIc, and FGFR4 to influence osteogenic and chondrogenic pathways by regulating the differentiation of osteoprogenitor cells into chondrocytes [170]. It downregulates genes responsible for ossification while promoting cartilage development and cell proliferation. FGF8 also drives inflammation by enhancing the production of inflammatory cytokines like interleukin-1β (IL-1β) and tumor necrosis factor-α (TNF-α), which further degrade the cartilage, resulting in conditions such as synovitis [170].

Disruption of FGF8 transcription can affect the gonadotropin-releasing hormone (GnRH) neuronal system, indicating that the protein provides essential trophic support for emerging GnRH neurons [174]. However, in GT1–7 neurons, FGF8 reduces GnRH mRNA expression, suggesting a dual role for FGF8: initially supporting the development of GnRH neurons but later needing to be downregulated to prevent dedifferentiation [174].

#### 2.4.2. FGF17

The role of FGF17 in the brain overlaps with that of FGF8 and FGF18, as the three of them support hindbrain and midbrain development [171,175]. Disruption of the FGF17 expression results in a decrease in the volume of the midbrain and a longer proliferation period in the vermis [175]. A study by Jen et al. showed that brain size regulation in mammalians by glypican-1 (Gpc1), a heparan sulfate proteoglycan, would be mediated through FGF17 and FGFR2 [176]. Furthermore, FGF17 plays a significant role in mammalian forebrain patterning [177]. Although FGF17-null mice survive, disturbances in the FGF17 functions may result in social and cognitive impairments [178–180]. FGF17 is thus of clinical significance in psychiatry. Yet, FGF17 is also detected in gynecological cancers such as ovarian and breast tumors [180].

#### 2.4.3. FGF18

During human embryonic development, FGF18 is first expressed in the cephalic mesenchyme and later expands into the mandibular mesenchyme, the perichondrium, and various other tissues by 8 weeks [181]. Between weeks 9 and 14 of gestation, its expression increases, particularly in airway smooth muscle cells, where it plays a key role in lung development. However, in adults, FGF18 is highly expressed in the heart and articular cartilage [181]. In the heart, FGF18 ameliorates cardiac hypertrophy and function by the activation of tyrosine-protein kinase FYN (FYN) and downregulation of NADPH oxidase 4 (NOX4) through FGFR3 binding [182].

The role of FGF18 in osteoarthritis has extensively been studied, and FGF18-based drugs such as Sprifermin are undergoing clinical trials for the treatment of knee osteoarthritis (KOA) [183–186]. FGF18 exerts its protective role against osteoarthritis by stimulating the MAPK pathways to enhance chondrocyte proliferation and support cartilage matrix production [186]. Intraarticular injection of FGF18 was also observed to increase cartilage thickness and joint space [184]. Lastly, FGF18 is involved in the progression of different cancers, such as colorectal, ovarian, lung, and breast cancer [181].

### 2.5. FGF9 Subfamily

FGF9 subfamily members are FGF9, FGF16 and FGF20 [177]. A unique feature in this subfamily is reversible homodimerization, which regulates the biological availability of FGF9 subfamily members. This mechanism of self-dimerization is postulated to play a role in ligand autoinhibition due to the downregulation of active monomer concentration that is needed for FGFR activation [187]. The FGF9 family members are mainly associated with embryonic development [188], neural differentiation and proliferation [189], sex determination [190], and organogenesis [191–193]. Further, they are associated with cancer [194] and other pathological conditions.

#### 2.5.1. FGF9

FGF9 is predominantly involved in male sex development, skeletal homeostasis, neural differentiation, and general homeostasis. FGF9 knockout mice displayed male-to-female sex reversal, highlighting the critical involvement of FGF9 in male sex fate [190]. Due to its significant functions in embryonic neural development and postnatal neural homeostasis, FGF9 is commonly known as the glia-activating factor [195].

Alterations in FGF9 levels and/or loss of function mutations of FGF9 are associated with pathological conditions, mainly attenuated sex development, skeletal and neural malignancies, and cancers. Bird et al. (2020) reported that Ser99Asn and Asn143Thr (equivalent to Arg62Gly in humans) FGF9 mutations in mice embryonic gonads contribute to drastic disorganizations in testis cords and partial reversion of XY sex. This was hypothesized to be due to the abrogation of FGF9–FGFR2c interactions in Ser99Asn and the combination of loss of FGF9–FGFR2c and FGF9–FGF9 homodimerization in Asn143Thr. Moreover, these same mutations were associated with craniosynostoses and multiple synostoses, which displayed cranial sutures fusion and limb joint fusion, respectively [196]. Moreover, dysregulation of FGF9 monomeric and homodimeric forms due to Asn143Thr mutation led to elbow knee synostoses [197]. An *Fgf9* promoter polymorphism, C712T, which led to decreased expression of FGF9, was associated with Sertoli cell-only syndrome in humans [198]. Further, reduced levels of FGF9 were associated with Wallerian degeneration upon nerve injury (impaired transformation of Schwann cells, making the blood–nerve barrier leaky) [195], ataxia defects [199], and Huntington’s disease [200,201]. In prostate cancer patients, the accumulation of FGF9 aggravated the cancer by promoting cancer cell proliferation and metastasis [202,203]. Further, overexpression of FGF9 was observed in colorectal cancer [204], gastric cancer [205], gastric and bladder cancer [206], Leydig tumor cells [207,208], hepatocellular carcinoma [209,210], lung squamous cell carcinoma [211], and testicular cancer [212].

In Wallerian degeneration, exogenous treatment of FGF9 rescued the disease phenotype and enhanced nerve fiber formation and the infiltration of macrophages [195]. Further, substantia nigra and mesencephalic neurons with FGF9 improved survival of dopaminergic neurons in vivo and in vitro [213]. Guo et al. (2021) reported that proteoliposome-mediated delivery of FGF9 in mice improved GABAergic neural survival and decreased the number of episodes of seizures; hence, it can be effective in treating people with epilepsy [214]. Moreover, adenoviral-mediated delivery of FGF9 after myocardial infarction improved systolic function and myocardial vascularization, showing a novel cardioprotective effect of FGF9 [215]. Later, Said et al. (2014) developed biodegradable polyester fibers loaded with FGF9 to ensure sustained delivery of FGF9 to treat patients with ischemic vascular disease. These fibers were able to release FGF9 for more than 28 days, which led to a significant increase in angiogenesis [216]. Carnosic acid was reported to be effective against depression by improving the FGF9–adiponectin–PPARγ signaling pathway [217].

In order to protect patients with lung squamous cell carcinoma, Wang et al. (2017) reported microRNA-mediated suppression of FGF9 using miR-372–3p [211], and Liang et al. (2020) reported that another miR-187 is effective against non-small-cell lung cancer progression by inhibiting FGF9 expression [218]. Moreover, Wang et al. (2020) described pharmacological inhibition of FGF9 overexpression using a novel FGF9-binding peptide using phage display screening. The antagonistic peptide abolished the FGF9–FGFR3c interaction at the D2–D3 linker region of FGFR3c and improved gastric and bladder cancer by inhibiting tumor cell proliferation and invasion. However, safety and specificity needed to be further evaluated to enhance the applicability of this peptide in clinical settings since FGF9–FGFR3c might be essential for other biological functions [206]. Cordycepin, a 3^′^-deoxyadenosine extracted from the fungus *Cordyceps sinensis*, was effective against testicular cancer by inhibiting FGF9 signaling [212].

Interestingly, a protective role of FGF9 is reported in improving lipid and glucose metabolism in the liver via FGFR3–PGE2-EP2/4 signaling in an autocrine fashion. FGF9 overexpression in hepatocytes downregulated the genes related to lipogenesis and stimulated gene pathways related to lipid oxidation, reducing lipid accumulation. The authors of the study predicted the novel function could help treat patients with non-alcoholic fatty liver disease (NAFLD) and related metabolic syndromes [219].

#### 2.5.2. FGF16

FGF16 is encoded on the X chromosome (Xq21.1) [220]. The mature peptide contains 207 amino acids without a cleavable N-terminal signal peptide sequence. FGF16 is a major player during embryonic heart development and maintenance of postnatal cardiac health. In murine embryonic heart, FGF16 promoted cardiomyocyte proliferation and heart development [191–193]. Myocyte enhancer factor 2 modulates the transcriptional activation of *Fgf16* (in neonatal rat cardiomyocytes) [192], and both epicardial and endocardial-derived FGF16 promote embryonic myocardial proliferation and differentiation [188].

Matsumoto et al. (2013) reported a potential protective role of FGF16 against cardiac hypertrophy and fibrosis [221] and the enhancement of cardiomyocyte proliferation during regeneration of the heart (post-natal period) [222,223]. Interestingly, the association of FGF16 with lipid metabolism was unraveled in the recent past. Overexpression of FGF16 led to stimulation of uncoupling protein 1 (UCP1) transcription and translation in inguinal white adipose tissues. Alterations in bile acid synthesis and reabsorption, as well as elevation of lipid excretion, were also observed. The effects were postulated to be mediated via FGFR1c [224]. Further, Huang et al. (2020) reported that FGF16 regulated preadipocyte differentiation in goat [225]. Further, in MCF10A cells (a breast cancer cell line), FGF16 promoted epithelial-to-mesenchymal transition by inducing aerobic glycolysis. GLUT3 and 6-phosphofructo-2-kinase/fructose-2,6-bisphosphate 4 mediated the FGF9-induced glycolysis, inferring that FGF16 can modulate glucose metabolism [226].

On the other hand, overexpression of FGF16 has been linked to breast cancer [226], lung cancer [194], and hepatocellular carcinoma [227]. X-linked recessive metacarpals 4/5 fusion is affiliated with *Fgf16* mutations in exon 3, namely, a truncation mutation c.474_477del, p.E158DfsX25 [228], and c.C535T;p.R179X [220]. To counteract the overexpression of FGF16 in cancer, the use of miR-520b (for lung cancer) [194] and miR-520f (for hepatocellular carcinoma) [227] microRNA therapies have been reported in the literature.

#### 2.5.3. FGF20

FGF20 is the last member of the FGF9 subfamily. It is encoded on chromosome 8p21.3-p22 [229]. The mature protein contains 211 amino acid residues. The association of FGF20 with embryonic development has been widely studied. Retinoic acid and erythropoietin regulate the expression of FGF9 subfamily members from the epicardium and endocardium, which subsequently induce myocardial proliferation [188]. Also, Zhang et al. (2010) reported differential regulation of FGF20-mediated endocardial and myocardial cardiomyocyte proliferation through Foxp1 transcription (elaborated in the FGF16 section of this review) [230]. Moreover, the redundant action of FGF9 and FGF20 promoted nephron progenitor cell stemness in mouse embryonic kidneys. Low levels of FGF20 led to nephron progenitor cell differentiation prematurely, and the complete absence of FGF20 resulted in renal agenesis, highlighting the importance of FGF20 in kidney development [231]. Trueb et al. (2013) reported that the developing kidney expressed a plethora of FGF members, including FGF20. FGF9 and FGF20 are expressed from metanephric mesenchymal cells and regulate the survival of progenitor cells in the kidney cortex [232]. Since these progenitor cells are absent in the adult kidneys, understanding FGF20-mediated stemness maintenance will unfold new treatment options to improve renal injury and regeneration. FGF20 is associated with cochlear and organ of Corti development. It was found to be expressed in the sensory epithelium of the ear during otic development, and it regulates the differentiation of cochlear progenitor cells into hair cells and supporting cells and the maintenance of the abundance of these cell populations [233]. Further, a significant affiliation of FGF20 was found with mammalian tooth development [234,235], dermal condensation of hair follicles during organogenesis [236,237], and dopaminergic neuron survival [189]. In mice, deletion of *Fgf20* led to complete hearing loss, suggesting FGF20 is associated with deafness [238].

In ovarian endometrioid adenocarcinoma, FGF20 was overexpressed and induced tumorigenesis [239]. A significant correlation has been found between FGF20 and Parkinson’s disease. The polymorphism rs12720208 (C>T) abrogated the 3^′^-UTR binding site for miRNA-433, which led to dysregulated overexpression of FGF20 signaling and overexpression of α-synuclein [240–242].

Since FGF20 was reported to stimulate dopaminergic neuron survival, its protective role in Parkinson’s disease should be clinically investigated. Delivery of exogenous FGF20 for a sustained time might improve the disease condition [189]. Moreover, FGF20-mediated maintenance of progenitor stem populations in the kidney and heart could be exploited to develop therapeutics for cardiac and renal regeneration upon injury [231,243]. Chen et al. (2021) reported that treatment of recombinant FGF20 after traumatic brain injury can stimulate the expression of proteins related to tight and adhesion junction formation and reduce inflammation and paracellular permeability, protecting blood–brain barrier integrity, suggesting a potential FGF20-mediated treatment paradigm [244]. Supporting this notion, Guo et al. (2021) also reported that FGF20 induced angiogenesis and neural and vascular repair, improving the recovery of cognitive function [245]. Further, Niu et al. (2018) reported a novel proteoliposome-mediated delivery of recombinant FGF20 guided by ultrasonography. The delivery of FGF20 via this method improved blood–brain barrier integrity in rats [246]. Moreover, human recombinant FGF20, velafermin (CG53135–05), was effective against mucositis in the gastrointestinal tract due to irinotecan-based chemotherapy and radiotherapy [247–249]. Importantly, a phase II randomized, double-blind, and placebo-controlled clinical trial has been performed to confirm the efficacy and safety of velafermin for oral mucositis (ClinicalTrials.gov ID—NCT00104065) [250].

## 3. Intracrine FGFs (iFGFs)

The intracrine subfamily members are often referred to as the fibroblast homologous factors (FHFs). This subfamily is composed of four members: FGF11 (FHF3), FGF12 (FHF1), FGF13 (FHF2), and FGF14 (FHF4) [251]. Although the members of this subfamily lack the signal peptide portion found in canonical FGFs, their core structure conserves high homology to canonical FGFs [251]. Similarly to paracrine FGFs, FHFs are constituted of 12 antiparallel β sheets arranged in a trefoil manner. The FHFs also exist in different forms as a result of alternative splicing in the N-terminus [251]. The ability of FGF11–14 to bind to heparin has also been found to be comparable to that of FGF10 with heparin. Yet, FHFs function intracellularly and do not activate FGFRs [177,251]. Instead, the primary effectors of the subfamily are the ion-gated channels [252]. Different reasons may underly the inability of FHF to bind to FGFRs, essentially the presence of Val157 in the receptor binding region of the protein and a structural conformation in the β8–β9 loop that would mismatch the FGFR binding site [251]. However, recent studies have demonstrated that, when administered, FHFs could signal in an FGFR-dependent manner but could also escape the intracellular region in a non-conventional secretion to signal different responses, such as cell proliferation and apoptosis inhibition [252,253].

### 3.1. FGF11 (FHF3)

The *Fgf11* gene is located on chromosome 17 [254], and the protein is highly expressed in the adrenal glands and the tonsils [255,256]. Its presence is also found in the testis and the stomach [256]. Current clinical interests in FGF11 focus mostly on the role of the protein in hypoxia [257,258]. A study by Nam et al. (2017) on whales suggests that hypoxia would induce FGF11 expression in the brain and heart of those subjects, increasing the tolerance of those subjects against the damage of low oxygen [257]. Its mechanism seems to be tightly linked to its interaction with hypoxia-inducible factor-1α (HIF-1α) [258]. FGF11 would also be capable of promoting the formation of “capillary-like tubes”, which would increase oxygen transport [257].

Another therapeutic importance of FGF11 is its potential role in adipogenesis in 3T3-L1. During the latest stages of differentiation, the level of FGF11 expression doubles in the cell. On the other hand, in the absence of FGF11, peroxisome proliferator-activated receptor gamma (PPARγ) levels decreased significantly, which hindered differentiation [259]. These findings show FGF11 as a new target in adipogenesis-related disorders. Lastly, FGF11 is also upregulated in lung cancer and may serve as a biomarker in cancer detection [260,261].

### 3.2. FGF12 (FHF1)

The *Fgf12* gene is located on chromosome 3 (3q29) [262] in humans and has two forms as a result of alternative splicing. FGF12a is the longest isoform, and FGF12b is the shortest [251,263]. FGF12 is present in different tissues, such as the kidneys, the pancreas, and the muscles, but it is more highly expressed in the nervous system, the heart, and the inner ears, where it communicates with the ion channels and ensures proper development [263]. FHFs have recently gained more interest, and the latest work on the subject has begun to shed more light on their mode of action and functions. Besides its role in inner ear development, FGF12 was found to inhibit radiation-induced apoptosis in mice models [253,264]. Interestingly, the study described that residues from 140 to 169 in FGF12 were responsible for such observation. These residues in the C-terminal would act as a cell-penetrating peptide domain to not only prevent apoptosis but also allow the introduction of other small peptides in the cell for therapeutic applications [264].

FGF12 ensures vascular smooth muscle cell plasticity by promoting differentiation into smooth muscle-like cells. These findings present a new target in vascular disease therapy, such as in atherosclerosis. Furthermore, Song et al. demonstrated in their study that a local administration of FGF12 in the carotid of injured rats could prevent the formation of intimal hyperplasia [265]. The absence of FGF12 also seems to impair the proper function of ion-gated channels while inducing liver fibrosis when it is overexpressed [266].

### 3.3. FGF13 (FHF2)

The *Fhf13* gene is located on q26 of the human X chromosome (Xq26) [267]. FGF13 is highly expressed in the developing brain and at higher concentrations than the other FHFs. It acts as a microtubule-stabilizing protein to support neuronal polarization and migration [268]. In mice, a low expression of FGF13 has led to impaired memory and learning capability [268]. Besides its role in brain development, FGF13 also positively affects sodium current in the heart. In mice models, the absence of FGF13 has resulted in the inability of the cardiac sodium channels to function properly and maintain conductibility at high temperatures [269,270]. Further understanding of this mechanism may be of clinical relevance in the treatment of Brugada syndromes [269].

### 3.4. FGF14 (FHF4)

The *Fhf14* gene is located on chromosome 17 [262], and the protein is highly expressed in the cerebellum, the hippocampus, and the olfactive bulbs [255]. FGF14 is of significant clinical importance for ataxia patients. Up to 60% of ataxia patients worldwide have exhibited a GAA repeat expansion in intron 1 of the *Fhf4* gene [271,272]. This condition, often referred to as GAA-FGF14 ataxia, may frequently cause cerebellar atrophy. Furthermore, FGF14 plays a crucial role in maintaining GABAergic activity in the CA1 hippocampal region. In the absence of FGF14, GABA synthesis and GABA expression are disrupted, resulting in impaired cognitive functions and brain disorders such as schizophrenia [273]. Alshammari et al. (2016) explained that the disruption in GABA signaling affects the excitatory–inhibitory balance, leading to reduced gamma oscillations often observed in schizophrenic patients [273]. Lastly, FGF14 acts as an apoptosis agent through the PI3K/AKT/mTOR signaling pathway in colorectal cancer [274].

## 4. Endocrine FGFs

### 4.1. FGF19 Subfamily

The FGF19 subfamily is composed of FGF19, FGF21 and FGF23. Unlike paracrine and endocrine FGFs, FGF19 subfamily members require an extra coreceptor, klotho, for their FGFR-mediated signaling due to their very poor affinity for FGFRs [2]. More specifically, FGF19 and FGF21 signaling is mediated through β-klotho, whereas FGF23 signaling occurs via α-klotho [4]. The subfamily members show differential affinity to different FGFRs [2]. FGF19-mediated physiological roles majorly arise from FGFR4, whereas FGF21 and FGF23 exert their functions mainly via the FGFR1c isoform. Being metabolic regulators, the FGF19 subfamily orchestrates a multitude of regulatory functions in the energy and nutrient metabolic homeostasis [1].

#### 4.1.1. FGF19

FGF19 is highly expressed in the small intestine, and its encoding gene is located on chromosome 11 (11q13.3) [275,276]. The protein contains a hydrophobic 20-residue region at the N-terminus, which functions as a signaling domain and is absent in the 21.8 kDa mature form [277]. FGF19 expression is signaled by the farnesoid X receptors, whose activation is triggered by the production of bile acids, occurring approximately two hours after feeding [278]. Once produced, FGF19 travels from the intestine to the liver, where it suppresses bile acid synthesis by inhibiting the rate-limiting enzyme cholesterol 7α-hydroxylase (CYP7A1) [278–280]. This regulatory function is critical in controlling bile acid levels and has clinical relevance in the treatment of bile acid-related disorders, such as chronic diarrhea, where treatments like Aldafermin (NGM282) have shown efficacy in reducing bile acid production [275,281].

Beyond its role in bile acid regulation, FGF19 is also vital in maintaining glucose and lipid homeostasis, particularly in adipose tissues [282,283]. This protein exerts its functions through interaction with β-klotho, a transmembrane protein, and its receptor FGFR4 [284,285]. Aided by its coreceptor, FGF19 activates the mitogen-activated protein kinase (MAPK) pathways, initiating a signaling cascade that downregulates hepatic gluconeogenesis, stimulates glycogen synthesis, and increases energy expenditure [275,286]. These properties make FGF19 a promising therapeutic target for metabolic conditions like type 2 diabetes [287]. Furthermore, FGF19’s ability to reduce fat tissue and improve lipid metabolism offers potential benefits in treating lipid-related disorders such as non-alcoholic fatty liver disease (NAFLD), non-alcoholic steatohepatitis (NASH), and obesity associated with diabetes [287–289].

Several FGF19 homologs have progressed to clinical trials, with Aldafermin (NGM282) showing encouraging results, particularly in liver diseases like NASH, where it has demonstrated improvements in liver fat content and fibrosis progression [290]. However, one significant challenge with FGF19 is its tumorigenic potential, especially for the hepatic cells [291]. To address this, research efforts have focused on modifying the N-terminal region of FGF19 to reduce its binding affinity to FGFR4 while preserving the C-terminal region responsible for binding to β-klotho and mediating the protein’s metabolic effects [292–294].

#### 4.1.2. FGF21

*Fgf21* resides on chromosome 19 (19q13.33) [295]. Key expression sites include hepatocytes, adipocytes, skeletal muscles, and pancreatic β-cells, each subject to diverse stimuli for FGF21 induction [296]. Upon secretion, human FGF21 consists of 209 residues, undergoing cleavage between the 28th and 29th residues to yield the active mature molecule [297]. FGF21 complements FGF19 with its distinct mechanisms of action while jointly contributing significantly to glucose and lipid homeostasis [2]. In the liver, fasting or a ketone-rich diet triggers peroxisome proliferator-activated receptor alpha (PPARα) and cAMP response element-binding protein (CREB) binding to the FGF21 gene to induce expression, while in adipocytes, peroxisome proliferator-activated receptor gamma (PPARγ) predominantly regulates expression [2]. The biological functions of FGF21 are mediated via FGFR and β-klotho interactions. Given this tissue-specificity, the regulatory factors and mechanisms governing FGF21 expression are intricate and multifaceted, with internal factors influenced by diet and alcohol consumption and external factors, such as temperature, impacting energy demand [296]. FGF21 is overexpressed in type 2 diabetes mellitus, obesity, non-alcoholic fatty liver disease (NAFLD), primary mitochondrial disorders, thyroid cancer, breast cancer, renal cancer, and endometrial cancer [2]. Several adipose-derived miRNAs regulate FGF21 levels in the body. The FGF21-mediated physiological functions are mediated through the ERK1/2-Akt pathway (in pancreatic β cells), Ras/Raf MAPK signaling, PPARα–FGF21 signaling, and FGF21–PGC-1α (in the liver during starvation) [2].

Owing to its significant role in glucose uptake, FGF21 is an attractive clinical target. The development of FGF21 analogs is well studied in the literature. LY2405319 is a more stable FGF21 analog (compared to wild-type FGF21) when administered subcutaneously into diet-induced obese mice, resulting in a reduction in plasma glucose levels and body weight [298]. Moreover, in diabetic rhesus monkeys, subcutaneous administration of LY2405319 led to improvement in glycemic and lipid parameters (i.e., glucose level, insulin, cholesterol, and triglyceride levels were reduced) [299]. These observations were consistent in humans with type 2 diabetes as well [300]. Further, PF-05231023 is another FGF21 analog with higher stability to regulate metabolic profiles in obese mice and humans [301–303]. Development of FGF21 antibody conjugates is also reported in the literature, where Giragosian et al. (2015) reported covalent conjugation of PF-05231023 with human IgG scaffold [303], Dirksen et al. (2018) reported a different FGF21 antibody conjugate using CVX-343 antibody scaffold [304], and Weng et al. (2018) developed PF-06645849, where they incorporated engineered glycans into previously discovered FGF21 analog PF-05231023 and fused it with Fc to attain higher proteolytic stability [305].

#### 4.1.3. FGF23

The *Fgf23* gene is located on chromosome 12p13.32 [306]. The immature transcript of FGF23 contains 251 amino acids with a molecular mass of 32 kDa [2]. During maturation, the N-terminal signaling peptide (24 amino acid residues) will be cleaved, giving rise to the biologically active form of FGF23 (227 amino acids; intact FGF23 or iFGF23), and the mature peptide hormone is released into the circulation [307]. O-glycosylation at Thr178 and/or phosphorylation at Ser180 stabilizes the FGF23 protein [307]. FGF23 is majorly produced by osteocytes and osteoblasts; however, its expression is reported in skeletal muscles, the brain, heart, kidney, mammary glands, stomach, and salivary glands [2]. FGF23 is a key mediator in phosphate homeostasis and vitamin D metabolism [2]. FGF23 expression is stimulated by 1,25-dihydroxyvitamin D, calcium, PTH, soluble klotho, and potentially by phosphorous. However, recent studies confirm that insulin, iron deficiency, erythropoietin, and pro-inflammatory cytokines (IL-6, TNFα) also stimulate FGF23 expression [307]. FGF23 is overexpressed in bone diseases such as autosomal dominant hyperphosphatemic rickets, X-linked hypophosphatemia (XLH), and tumor-induced osteomalacia (TIO), as well as in diabetic nephropathy, acute and chronic kidney diseases, cardiovascular conditions such as coronary artery disease and atherosclerosis, and inflammatory disorders [1]. The interactions between FGF23 and its specific coreceptor, alpha klotho with FGFR, will activate MAPK/ERK signaling, the MAPK–FRS2 pathway, and the PLCγ1–calcineurin–NFAT pathway [2]. Burosumab (KRN23, Crysvita) is a monoclonal IgG1 antibody treatment for X-liked hypophosphatemia (Trial No. NCT03920072, phase 3b) and tumor-induced osteomalacia (Trial No. NCT02304367), and it was approved by the FDA in 2018. This anti-FGF23 therapy was successful in reducing the excess FGF23 levels seen in phosphate-wasting disorders by promoting phosphate reabsorption in the kidney, which led to a reduction in plasma phosphate levels by inhibiting FGF23–FGFR–aKL signaling [308]. Small molecule inhibitors are also developed against FGF23-overexpression-mediated signal dysregulation (e.g., INCA-6 (abrogated FGF23–FGFR4–calcineurin–NFAT signaling pathway) [309] and ZINC1240912 (FGF23-alpha klotho-ERK 1/2 signaling)) [310]. Several studies reported that the administration of FGF23 C-tail ameliorated functional and morphological abnormalities of diabetic nephropathy, reducing renal inflammation and fibrosis [311]. The C-tail fragment can bind to the FGFR1c–alpha klotho complex; however, it cannot induce signaling. Thus, the administration of FGF23 C-tail abolishes FGF23–alpha klotho-mediated signaling by acting as an internal inhibitory peptide. Further, exogenous delivery of FGF23 C-tail to rats and mice increases serum phosphate levels in vivo, raising the possibility of using it in a therapeutic setting to treat phosphate-wasting disorders [311]. However, the half-life of the 72-amino acid residue-long peptide was reported to be very short (10 min), thus restricting its applicability. Hence, a fusion protein, FGF23 C-tail-Fc, was developed to obtain FGF23 antagonism for a sustained time. Repeated injection of FGF23-C-tail Fc in Hyp mice, a preclinical model of XLH, increases cell surface abundance of NaPi transporters, normalizes phosphate excretion, and improves bone architecture [312]. A novel FGF23-bonding peptide corresponding to FGF23 180–205 (referred to as 23-b6) was discovered using phage display technology and showed high homology to D3 of FGFR1c. Treating with 23-b6 increased phosphate uptake in opossum kidney cells by blocking the Erk pathway and upregulating NaPi-2a and 2c expression, suggesting that 23-b6 is a potent antagonist of FGF23 [313].

A summary of the primary biological functions of fibroblast growth factors (FGFs), along with the diseases associated with alterations in FGF transcription and expression, is provided in Table 1.

A summary of various strategies to treat FGF-related disorders, that had been published in literature, is provided in Table 2.

Key examples of FGF analogs and antagonists tested in clinical trials are provided in Table 3.

## 5. Biomedical Concerns of FGF-Mediated Therapy

FGF-mediated therapeutic strategies hold significant promise in the development of therapies against many disorders. However, several ethical and biomedical concerns warrant careful consideration due to the intricate nature of FGF–FGFR signaling pathways and the potential detrimental side effects during therapeutic targeting [336]. Off-target effects are a major safety concern related to FGF-based therapy since FGF signaling is not restricted to a single tissue or organ system. Moreover, targeting FGFRs can give rise to a plethora of detrimental side effects disrupting normal cellular processes, which can subsequently give rise to dysregulated cell proliferation, tissue fibrosis, and cancer [337]. Another safety concern is the long-term consequences, where chronic dysregulation of normal growth and tissue repair leads to chronic inflammation or fibrosis. Moreover, such long-term consequences of FGF-based therapies are not fully understood yet. Hence, long-term toxicity or complications might not be evident at the time of treatment [336].

## 6. Conclusions

FGF–FGFR signaling is crucial in development and pathogenesis. An in-depth understanding of the interactions of FGF-mediated signaling pathways with other signaling pathways, genetic regulation of FGFs, and associated molecular mechanisms will be necessary to implement new approaches targeted to a plethora of FGF-mediated genetic, metabolic, and degenerative disorders, cancer, and wound healing. Here, we described biological functions, disorders, and therapeutic interventions based on each FGF. However, due to their widespread distribution, selective modulation of FGF-mediated pathways is challenging. Recent advances in the development of small molecule inhibitors, monoclonal antibodies that abrogate FGF–FGFR dysregulated signaling, gene therapy, and mechanisms for targeted delivery of FGFs are discussed.

## Figures and Tables

**Table 1.** Summary of main biological functions and diseases of FGFs.

Subfamily	FGF	Main Functions	Diseases
FGF1 (Paracrine)	FGF1	Cell proliferation and differentiation Angiogenesis [7] Wound healing [16] Neural development [7] Increase insulin sensitization and glucose uptake [19] Adipose remodeling [19]	Cancer (non-small-cell lung cancer [33], colorectal cancer [34], gastric cancer [35], breast cancer [13], ovarian cancer [13], and pancreatic cancer [31])
	FGF2	Angiogenesis [43] Wound healing [43] Cartilage and tendon repair [48]	Osteoarthritis [49] Cancer (squamous lung cancer [45], hepatocellular carcinoma [47], and non-small-cell lung cancer [46])
FGF4 (Paracrine)	FGF4	Limb development [55] Maintenance of pluripotency [59] Macrophage survival [61]. Wound healing [63]	Liver diseases (non-alcoholic fatty liver disease and autoimmune hepatitis) [61]
	FGF5	Hair growth regulation [73] Stress alleviation [75] Muscle development [77]	Lung cancer [77,78]
	FGF6	Angiogenesis [81] Myogenesis [81–84] Cardiac repair [84]	Bladder cancer [81]
FGF7 (Paracrine)	FGF3	Ear [90], neural [94], brain [92], cardiovascular [93], and head skeleton development [97]	Auditory impairments/ear malformations [98] Cancer (Kaposi's sarcoma [99], hepatocellular carcinoma [100], non-small-cell lung disease [102], and breast cancer)
FGF7 (Paracrine)	FGF7	Wound healing [111,112] Hair growth [113] Bone development [116] and glandular development (mammary gland [314] and submandibular glands [315])	Mandible defects [116] Cancer (gastric cancer [316] and leukemia [317])
	FGF10	Glandular development (mammary glands [318], salivary glands [136]), lung [138], and neural development [94] Cardiac regeneration [319]	Lung and limb agenesis [320] Glandular defects (e.g., Lacrimo-auriculo-dento-digital syndrome [138] and aplasia of the lacrimal and salivary glands [138]) Cancer (cholangiocarcinoma [148])
	FGF22	Neural [152], brain [157], and ear development [155] Spinal cord remodeling [165] Liver regeneration [168]	Auditory defects/deafness [155]. Depression [163] Cancer (ovarian cancer [159], pancreatic cancer [161], and lung cancer [160])
FGF8 (Paracrine)	FGF8	Limb development [170] Brain development [171,172] Bone and cartilage development [170]	Neural defects [174]
	FGF17	Brain development [171,175]	Cognitive impairments [178–180] Cancer (breast cancer and ovarian cancer [180])
	FGF18	Brain development [181] Heart and cartilage development [181]	Osteoarthritis [183–186]. Cancer (colorectal cancer, ovarian cancer, and breast cancer [181])
FGF9 (Paracrine)	FGF9	Neural differentiation [195] Male sex development [190] Skeletal development and homeostasis [196]	Sertoli cell-only syndrome [198] Huntington's disease [200] Craniosynostoses [196] Cancer (prostate [202,203], colorectal [204], gastric cancers [205], Leydig tumor [207,208], and hepatocellular carcinoma [209,210])
	FGF16	Embryonic heart development [191–193] Inner ear development [321,322] Brain development [323] Oocyte development [324]	Cancer (breast [226] and lung cancer [194] and hepatocellular carcinoma [227])
	FGF20	Embryonic and post-natal heart development [188], kidney [231], cochlear and organ of Corti [233], and tooth development [234,235] Cardiac and kidney regeneration [231,243]	Deafness [238]. Parkinson's disease [189]
Intracrine FGFs	FGF11	Glandular development [255,256] Adipogenesis [259]	Lung cancer [260,261]
	FGF12	Maintenance of vascular muscle plasticity [265]	Atherosclerosis [265]
	FGF13	Brain development Cognitive function Maintenance of heart sodium channel [269,270]	Memory and learning ability impairments [268] Brugada syndromes [269]
	FGF14	GABAergic neuron maintenance [273].	Ataxia [271,272] Cerebelar atrophy [271,272] Schizophrenia [273] Colorectal cancer [274]
FGF19 (Endocrine)	FGF19	Regulation of bile acid synthesis [278] Glucose and lipid homeostasis [282,283]	Bile acid disorders [275,281] Type 2 diabetes [287] Non-alcoholic fatty liver disease [287–289] Non-alcoholic steatohepatitis [287–289] Obesity [287–289]
	FGF21	Glucose and lipid homeostasis [2]	Type 2 diabetes [296] Non-alcoholic fatty liver disease [2] and Non-alcoholic steatohepatitis [2] Obesity [2], cancer (breast cancer, thyroid cancer, renal cancer, and endometrial cancer) [2]
	FGF23	Vitamin D and phosphate level homeostasis	Tumor-induced osteomalacia [1] X-linked hypophosphatemia [1] Kidney diseases heart disorders [1]

**Table 2.** Summary of selected therapeutic strategies against FGF-related disorders.

Subfamily	FGF	Strategy	Therapeutic Application	Disease
FGF1	FGF1	FGF1-loaded lipid nanoparticles	Promoted microvascular sprouting	Diabetic foot ulcers [21]
		Gene therapy	Reduced inflammation [22] and promoted neovascularization [23]	Improved neuropathic pain due to chronic constriction injury [22]
		Hydrogels	Accelerated wound healing [24], restored thickness of the uterine wall, and abundance of uterine glands [25]	Wound healing [24] and endometrial injury and fibrosis [25]
		FGF1^ΔHBS^ (K127D/K128E/K133V)	Reduced the size of the infarction, restored contractile and relaxation rhythms [27], and decreased apoptosis and oxidative stress in primary cardiomyocytes [28] and in podocytes [29]	Cardiac ischemia-reperfusion injury and myocardial infarction [27], cardiac toxicity during chemotherapy [28], diabetic nephropathy [29]
	FGF2	FGF1^ΔHBS^-FGF21^C-tail^ chimera	Elevated glycemic control and improved insulin resistance	Diabetes [32]
		Antagonistic peptide for FGF2 (P8)	Reduced FGF2-mediated tumor cell proliferation without cytotoxic consequences	Cancer [50]
		Cidofovir	Enhanced apoptosis and FGF2-mediated cancer cell proliferation	Vascular tumors [51]
		RBM-007 aptamer	Reduced dysregulated neovascularization	Blinding diseases (e.g., age-related macular degeneration) [52]
		Collagen-heparin scaffolds with FGF2 (and VEGF)	Induced angiogenesis	Ischemic heart disease, diabetic foot ulcers [54]
FGF7	FGF3	FP16 (FGF3 antagonist peptide)	Abrogated FGF3-FGFR2 interactions and inhibited FGF3-mediated cancer cell proliferation	Breast cancer [104]
		LY2874455 (selective pan-FGFR inhibitor) combined with abemaciclib	Inhibited FGF3/FGF4/FGF19/CCND1 amplification	Non-small-cell lung cancer [102]
	FGF7	Single-domain antibodies against FGF7	Inhibit FGF7-FGFR signaling	Cancer [127]
		FGF7-releasing galactosylated poly (DL-lactide-co-glycolic acid) particles	Improved liver cell proliferation and hepatic metabolism	Diabetes [120]
		Palifermin (Kepivance^™^, human recombinant FGF7)	Alleviate severe oral mucositis	Gastrointestinal mucositis during chemotherapy [122–126]
		Neutralizing antibodies against FGF7	Inhibition of FGF7 signaling	Ovarian cancer [132]
	FGF10	Infigratinib (FGFR inhibitor)	Suppressed FGF10-FGFR2b mediated tumor metastasis	Cholangiocarcinoma [148]
		FGF10-loaded poly (lactic-co-glycolic acid) microsphere particles	Sustained secretion of FGF10 at the wound site stimulated epithelial cell proliferation, migration, and differentiation Attenuated ER stress	Wound healing [150]
	FGF22	Exogenous FGF22	Promoted synapse formation through FGF22-FGFR1b and FGF10-FGFR2b interactions	Promote spinal cord remodeling upon injury [165]
		Gene therapy	Alleviated ER stress and ER-stress-induced apoptosis of neuronal cells [165,166] and stimulated the proliferation and survival of A6-expressing hepatocytes [168]	Promoted axon regeneration during recovery from spinal cord injury [165,166] and liver regeneration [168]
		Recombinant FGF22	Facilitated calcium influx into the inner hair cells and promoted ribbon synapse formation	Auditory impairments [169]
FGF4	FGF4	Alferminogene tadenovec	Increased angiogenesis (phase 3 clinical trial)	Myocardial Ischemia (in women) [69]
		Phagocytes transfected with FGF4 gene with biodegradable gelatin complex	Increased angiogenesis	Myocardial Ischemia [70]
		Exosomes derived from mmu_circ_0001052-adipose-derived stem cells	Increased angiogenesis	Diabetic foot ulcers [71]
	FGF5	CRISPR/Cas9 mediated Fgf5 gene knockout	Increased fine wool and active hair follicle density	Hair growth [75,325]
		Monoterpenoid family compounds	Improved hair density	Hair growth/hair loss [79]
		Cholesterol-modified siRNA	Improved hair density	Hair growth/hair loss [80]
	FGF6	Recombinant FGF6	Promoted soleus regeneration	Myogenesis [86,326]
FGF8	FGF18	Gene therapy	Increased chondrocyte number	Cartilage repair [327]
		Hydrogel	Increased osteogenic progenitor cell number and cell infiltration	Improved bone repair [328]
		Subcutaneous injection of FGF18	Induced anagen from telogen phase of hair follicles	Hair growth [329]
		Sprifermin (Recombinant FGF18)	Increased chondrocyte proliferation	Osteoarthritis [330]
FGF9	FGF9	Exogenous FGF9	Enhanced nerve fiber formation and infiltration macrophages	Wallerian degeneration [195]
		Proteoliposome-mediated delivery of FGF9	Improved GABAergic neural survival and decreased the number of episodes of seizures	Epilepsy [214]
	FGF16	miR-520b	Inhibited FGF16 gene expression	Lung cancer [194]
		miR-520f	Inhibited FGF16 gene expression	Hepatocellular carcinoma [227]
	FGF20	Proteoliposome-mediated delivery of recombinant FGF20 guided by ultrasonography	Improved blood-brain barrier integrity	Traumatic brain injury [246]
		Velafermin (CG53135–05) (human recombinant FGF20)	Reduced mucositis in the gastrointestinal tract	Protects against Irinotecan-based chemotherapy and radiotherapy [247–249]
FGF19	FGF19	Aldafermin (NGM282)	Improved liver fat content and fibrosis progression	NASH [290]
	FGF21	LY2405319 (an FGF21 analog)	Reduced plasma glucose levels and body weight	Diabetes and Obesity [298]
	FGF23	Burosumab	Abolish FGF23-FGFR-aKL signaling	X-linked hypophosphatemia [308]

**Table 3.** Selected completed clinical trials testing FGF-based therapeutics.

FGF	Trial Identifier	Disease/Condition	Intervention	Phase	Outcome
FGF1	NCT00425178	Diabetic or venous stasis ulcers	Topical administration of FGF1	Phase 1	Not available
FGF1	NCT04520321	Corneal endothelial dystrophy	TTHX1114 (an engineered form of FGF1) via intracameral delivery	Phase 1/2	An injection volume of 10 mcL of TTHX1114 reported to be safe [331]
FGF2	NCT02307916	Tympanic membrane perforation	FGF-2 in a pledget of gelatin foam (topical application)	Phase 2	Not available
FGF2	NCT02337166	Periodontal wound healing	rFGF2 in sodium hyaluronic acid carrier	Phase 1/2	Significant reduction of probing depth, probing attachment level, and shallower residual probing depth were observed compared to controls [332]
FGF4	NCT00346437	Angina pectoris	Intracoronary infusion of adenoviral gene for FGF4 (Ad5FGF4)	Phase 2/3	Improved myocardial perfusion [333]
FGF7	NCT00101582	Oral mucositis	rFGF7 (Palifermin)	Phase 3	Not available
FGF7	NCT00094861	Dysphagia in NSCLC	rFGF7 (Palifermin)	Phase 2	Not available
FGF7	NCT00031148	Graft vs host disease after allogenic hematopoietic stem cell transplantation	rFGF7 (Palifermin)	Phase 1/2	Reported to be safe during allogenic hematopoietic stem cell transplantation without significant effects on engraftment [334]
FGF18	NCT01919164	Osteoarthritis	rFGF18 (Sprifermin)	Phase 2	Regeneration of knee cartilage in sprifermin-treated group compared to controls
FGF19	NCT03059537	Bile acid malabsorption	Chenodeoxycholic acid	Phase 4	Not available
FGF19	NCT02443116	NASH	NGM282 (Aldafermin)	Phase 2	Reduced liver fat, inflammation, and fibrosis in Aldafermin treated subjects
FGF21	NCT02413372	NASH	BMS-986036 (Pegbelfermin)—FGF21 analog; subcutaneous administration	Phase 2	Reduced liver fat content in NASH subjects [335]
FGF21	NCT01673178	T2DM	PF-05231023 (FGF21 analog)	Phase 1	Lowered triglycerides in obese subjects with or without T2DM
FGF23	NCT02750618	XLH	Burosumab (FGF23 blocking antibody)	Phase 2	Increased serum phosphate levels

Information was extracted from https://clinicaltrials.gov (accessed on 20 November 2024). Note that rFGF: recombinant FGF; NSCLC: non-small-cell lung cancer; NASH: non-alcoholic steatohepatitis; T2DM: type 2 diabetic mellitus; and XLH: X-linked hypophosphatemia.

## Data Availability

No new data were created or analyzed in this review. Data sharing is not applicable to this article.
